# Adherens Junctions: Guardians of Cortical Development

**DOI:** 10.3389/fcell.2020.00006

**Published:** 2020-01-28

**Authors:** Lenin Veeraval, Conor J. O’Leary, Helen M. Cooper

**Affiliations:** Queensland Brain Institute, The University of Queensland, Brisbane, QLD, Australia

**Keywords:** cortical development, radial glia, adherens junctions, cortical malformation, ependymal cell, apicobasal polarity, actin cytoskeleton, cadherin

## Abstract

Apical radial glia comprise the pseudostratified neuroepithelium lining the embryonic lateral ventricles and give rise to the extensive repertoire of pyramidal neuronal subtypes of the neocortex. The establishment of a highly apicobasally polarized radial glial morphology is a mandatory prerequisite for cortical development as it governs neurogenesis, neural migration and the integrity of the ventricular wall. As in all epithelia, cadherin-based adherens junctions (AJs) play an obligate role in the maintenance of radial glial apicobasal polarity and neuroepithelial cohesion. In addition, the assembly of resilient AJs is critical to the integrity of the neuroepithelium which must resist the tensile forces arising from increasing CSF volume and other mechanical stresses associated with the expansion of the ventricles in the embryo and neonate. Junctional instability leads to the collapse of radial glial morphology, disruption of the ventricular surface and cortical lamination defects due to failed neuronal migration. The fidelity of cortical development is therefore dependent on AJ assembly and stability. Mutations in genes known to control radial glial junction formation are causative for a subset of inherited cortical malformations (neuronal heterotopias) as well as perinatal hydrocephalus, reinforcing the concept that radial glial junctions are pivotal determinants of successful corticogenesis. In this review we explore the key animal studies that have revealed important insights into the role of AJs in maintaining apical radial glial morphology and function, and as such, have provided a deeper understanding of the aberrant molecular and cellular processes contributing to debilitating cortical malformations. We highlight the reciprocal interactions between AJs and the epithelial polarity complexes that impose radial glial apicobasal polarity. We also discuss the critical molecular networks promoting AJ assembly in apical radial glia and emphasize the role of the actin cytoskeleton in the stabilization of cadherin adhesion – a crucial factor in buffering the mechanical forces exerted as a consequence of cortical expansion.

## Introduction

Uniquely human attributes such as consciousness, creativity and language as well as other higher order functions, including sensory perception, learning and memory arise as a consequence of the enormous array of excitatory pyramidal neurons that populate the six-layered neocortex. As a general principle, neurons of a given subtype are associated with a specific cortical layer (Molyneaux et al., 2007; Fame et al., 2011). For example, the corticospinal motor neurons which project their axons subcerebrally to the spinal cord are the principal pyramidal subtype of layer 5. In contrast, cortical projection neurons predominantly populate layers 2/3 and send their axons across the corpus callosum to the contralateral hemisphere. However, in reality, cortical architecture is considerably more complex as a variety of subtypes exist within each layer, and conversely, one subtype can be found in multiple layers (O’Leary and Koester, 1993; Kasper et al., 1994; Molyneaux et al., 2007; Fame et al., 2011). This elaborate cytoarchitecture is fundamental to the establishment of the neuronal circuitry which dictates the extent and quality of information flow across the cortex and between the cortex and subcortical regions. Our ability to effectively interact with the world is therefore dependent on the fundamental cell biological processes regulating the birth of new neurons and their subsequent migration to their predetermined cortical layer.

**FIGURE 1:**
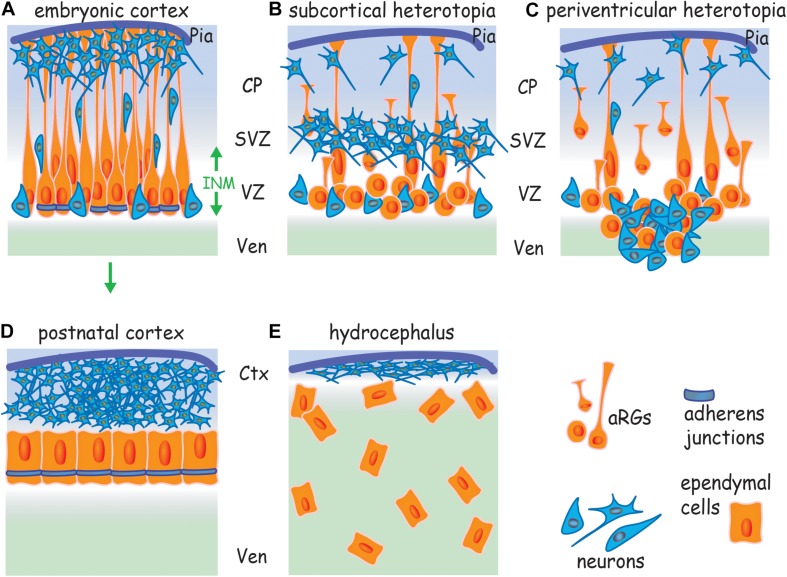
Cortical malformations arise from a failure to assemble AJs. **(A)** Apicobasally polarized RGs undergo symmetric divisions to expand the progenitor pool and then switch to asymmetric division, generating a new apical RG and a neuron or intermediate progenitor. Neurons undergo radial migration along basal processes to populate the cortical plate. AJs encircle the apical RG at the border of the apical and lateral membranes. Failure to assemble AJs and the subsequent loss of RG polarity leads to **(B)** subcortical band heterotopia due to the retraction of basal processes, and **(C)** periventricular heterotopia due to the retraction of basal processes and disruption of the ventricular wall. **(D)** Ependymal cells derive from apical RGs (arrow) and form an apicobasally polarized epithelium. **(E)** Hydrocephalus arises from a failure to establish a cohesive ependymal layer due to the disruption of AJs in apical RGs and ependymal cells. Ctx, cortex; CP, cortical plate; INM, interkinetic nuclear migration; SVZ, subventricular zone; Ven, ventricle; VZ, ventricular zone.

The neural progenitors that give rise directly or indirectly to all pyramidal neurons in the neocortex are classified as apical radial glia (apical RGs) and comprise the pseudostratified neuroepithelium lining the embryonic ventricles (Figure 1A) (Kriegstein and Alvarez-Buylla, 2009; Paridaen and Huttner, 2014; Taverna et al., 2014). The highly apicobasally polarized morphology of apical RGs is a mandatory requirement for successful cortical development as it governs the mode of cell division, neuronal production, specification and migration, and the integrity of the ventricular wall. Adherens junctions (AJs), the sites of cadherin-mediated cell–cell adhesion, play an obligate role in the induction and maintenance of apical RG polarity and are thus pivotal determinants of progenitor function and the establishment of a functional neocortex (Stocker and Chenn, 2015; Bustamante et al., 2019). Throughout the developmental period AJs undergo constant remodeling as RGs divide, new progenitors re-establish adhesion and their progeny detach from the neuroepithelium. Concomitantly, it is essential that RG junctions resist the mechanical stresses exerted as a consequence of these cellular behaviors as well as the forces incurred from increasing cerebrospinal fluid (CSF) volume and flow.

Junctional instability leads to the collapse of apical RG morphology, disruption of the ventricular surface and cortical lamination defects due to failed neuronal migration (Kadowaki et al., 2007; Cappello et al., 2012; Gil-Sanz et al., 2014; O’Leary et al., 2017). As in all epithelia, adhesive strength is determined by reciprocal interactions between the cadherins and the actin cytoskeleton which also regulates junctional tension through actomyosin contractility (Priya and Yap, 2015; Yap et al., 2017). It is now clear that junctional failure underpins the etiology of cortical malformations where the inability to assemble AJs leads to reduced neuronal production, the accumulation of neurons in ectopic positions (neuronal heterotopias), incorrect cortical layering and disruption of the ventricular wall. Mutations responsible for inherited cortical malformations have been identified in genes known to control junction formation (Fox et al., 1998; Sheen et al., 2003; Ferland et al., 2009; Lian and Sheen, 2015). These cortical malformations have profound consequences for survival and the individual’s quality of life. Therefore, the molecular pathways that promote cadherin adhesion and actin remodeling are crucial factors safeguarding the fidelity of cortical development.

During late embryonic and early postnatal life apical RGs transform into ependymal cells which line the ventricles throughout the adult central nervous system. Ependymal cells form an apicobasally polarized, multi-ciliated epithelium that acts as a bidirectional barrier to transport CSF components between the ventricle and the brain parenchyma (Del Bigio, 2010). The establishment of cadherin-based AJs and a polarized ependymal morphology is essential for barrier function. Hydrocephalus, a prevalent neurodevelopmental disorder associated with neonatal lethality, severe intellectual impairment and motor dysfunction, is characterized by the dilation of the lateral ventricles and reduction in cortical tissue volume (ventromegaly) resulting from excessive CSF (Kousi and Katsanis, 2016). Failure to establish a cohesive ependymal layer in the neonatal telencephalon due to the disruption of AJs in ependymal cells and their predecessors, the apical RGs, is causal for hydrocephalus (Dominguez-Pinos et al., 2005; Rodriguez et al., 2012; Guerra et al., 2015). Moreover, the occurrence of hydrocephalus is often coincident with neuronal heterotopias, indicating that these cortical malformations share a common etiology – the loss of AJs (Sheen et al., 2004; Rodriguez et al., 2012; Jimenez et al., 2014; Guerra et al., 2015).

In this review, we focus on the mechanisms by which AJs ensure successful cortical development and reflect on how our knowledge of apical RG and ependymal junction biology has led to a deeper understanding of the aberrant molecular and cellular processes contributing to devastating cortical malformations. We will explore the pivotal animal studies that have revealed important insights into the role of AJs in maintaining apical RG and ependymal morphology and function, and discuss the important molecular interactions underpinning AJ assembly. We highlight the reciprocal interactions between junctions and the epithelial polarity complexes which govern the establishment of RG apicobasal polarity. In addition, we discuss the role of the actin cytoskeleton in the stabilization of cadherin adhesion – a crucial factor in buffering the mechanical forces exerted as a consequence of cortical expansion. We begin, however, with a brief summary of the key features of cortical development. For more in-depth reviews on this topic see Paridaen and Huttner (2014), Taverna et al. (2014), Lodato and Arlotta (2015), Hansen et al. (2017), and Huttner and Wieland (2019).

### A Brief Summary of Cortical Development

In the early vertebrate embryo the brain and spinal cord arise from the pseudostratified neuroepithelial stem cells of the neural tube. Corticogenesis begins in the dorsal telencephalon when these cells transform into apical RGs which retain their apicobasal polarity throughout development by anchoring their basal end feet to the pial extracellular matrix and their apical end feet to the ventricular surface (Figure 1A) (Kriegstein and Alvarez-Buylla, 2009; Paridaen and Huttner, 2014). Production of cortical neurons is temporally restricted, with deep layer 5/6 neurons born in the early phase of corticogenesis and upper layer 2/3 neurons generated in the later developmental period (Molyneaux et al., 2007; Fame et al., 2011). Initially, apical RGs undergo symmetric self-renewing divisions to expand the progenitor pool and then switch to asymmetric neurogenic divisions, generating a progenitor and a neuron which is destined to populate deep layers 5/6 (Miyata et al., 2004; Huttner and Kosodo, 2005; Konno et al., 2008; Paridaen and Huttner, 2014; Oberst et al., 2019). As corticogenesis proceeds the generation of neurons directly from asymmetric RG division ceases, and instead, an array of diverse intermediate progenitor populations are produced (Farkas and Huttner, 2008; Kowalczyk et al., 2009; Postiglione et al., 2011). These secondary progenitors then undergo neurogenic division above the ventricular zone (i.e., in the subventricular zone; Figure 1A) to give rise predominantly to upper layer pyramidal neurons. It is the evolutionary expansion of these apical RG-derived intermediate progenitor populations that is responsible for the extensive production of pyramidal neurons that gives rise to the gyrencephalic architecture of the human neocortex. The evolution and cell biology of these fascinating progenitor populations have been comprehensively reviewed by others (Smart et al., 2002; Reillo et al., 2011; Betizeau et al., 2013; Kelava et al., 2013; Huttner and Wieland, 2019). It should be further noted that the mechanical gradients generated in the embryonic cortical tissue and the biomechanics of cortical folding associated with the expansion of the gyrencephalic cortex do not involve apical RGs. Instead, cortical folding arises as a consequence of the massive accumulation of neurons within the cortical plate. The vast majority of these neurons are generated from the basal radial glia (intermediate progenitors) within in the subventricular zone (Striedter et al., 2015; Llinares-Benadero and Borrell, 2019).

Due to the requirement to accommodate large numbers of progenitors within a confined space apical RGs undergo a specialized form of cell division, interkinetic nuclear migration, in which their nuclei migrate toward the pial surface during the G1 phase of the cell cycle (Figure 1A) (Sauer, 1935; Murciano et al., 2002; Taverna and Huttner, 2010). S-phase is completed at the ventricular-subventricular interface before the nucleus returns to the ventricular surface to undergo mitosis. During interkinetic nuclear migration the apical RG maintains its attachment to the ventricular surface, thereby ensuring that the primary cilium and associated centrosomes are retained at the apical membrane throughout the cell cycle (Taverna and Huttner, 2010; Das and Storey, 2014). This distinctive mode of cell division demarcates the proliferative ventricular zone, endows the neuroepithelium with its pseudostratified cytoarchitecture and is a prerequisite for the expansion of the progenitor pool.

As new-born neurons populate the emerging cortical plate, the basal processes of the apical RGs increase in length (Figure 1A) (Miyata et al., 2001; Noctor et al., 2002). It has been estimated that the lateral membrane of the basal process comprises the vast majority of the RG plasma membrane, whereas the apical (ventricular) membrane encompasses only 2% of the total surface area (Kosodo et al., 2004). Attachment of the basal end feet to the pial extracellular matrix is dependent on the α_6_β_1_, α_3_β_1_ and α_V_β_3_ integrins and their deletion leads to the detachment of RG basal processes (Haubst et al., 2006; Jeong et al., 2013). Laminin/β_1_ integrin interactions are also required to anchor the apical end feet to the ventricular surface (Loulier et al., 2009). Blocking these interactions induces RG detachment, retraction of the basal process and cortical layering defects.

Retention of the highly polarized RG morphology is essential for the expansion and lamination of the six-layered cortex (Nadarajah et al., 2001; Farkas and Huttner, 2008; Kriegstein and Alvarez-Buylla, 2009). Each layer of the cortex is sequentially generated as waves of neurons migrate along the basal processes (radial migration, Figure 1A) to populate their predetermined cortical layer. In the early phase of corticogenesis the deep layer pyramidal neurons arising from RG asymmetric neurogenic divisions dissociate from the ventricular surface, attach to the basal processes and undergo radial migration to establish layers 5/6. Subsequently, the upper layer neurons, generated by intermediate progenitor neurogenic divisions, also employ radial migration along the basal processes in order to migrate through the pre-established deep layer neurons.

In summary, the highly polarized morphology of apical RGs is a mandatory prerequisite for cortical development as it directly influences the expansion of the RG progenitor pool, the decision to exit the cell cycle, acquisition of neuronal identity and cortical lamination, all of which are tightly synchronized in space and time. As in all epithelia, cadherin-based AJs play an obligate role in the induction and maintenance of RG apicobasal polarity and are therefore essential to the fidelity of cortical development.

### Adherens Junctions Preserve Cortical Architecture

Adherens junctions encircle the RG at the border of the apical and lateral membranes within the apical end feet (Figure 1A). In contrast to other epithelia, apical RGs do not have tight junctions and thus rely on cadherin-based AJs to maintain polarity and tissue cohesion (Aaku-Saraste et al., 1996). Cortical malformations such as subcortical band heterotopia in which neurons accumulate below the cortical plate and periventricular heterotopia, characterized by ectopic neuron-containing nodules protruding into the lateral ventricle (Figures 1B,C) have previously been attributed to defects in neuronal motility. However, recent studies in rodents and humans have recognized that the principal mechanism contributing to the etiology of these malformations is the disruption of apical RG morphology as a consequence of the inability to assemble AJs (Rodriguez et al., 2012; Lian and Sheen, 2015). Moreover, mutations in genes known to control junction formation have been shown to be causative for inherited forms of these cortical malformations (Fox et al., 1998; Sheen et al., 2003; Ferland et al., 2009), reinforcing the concept that apical RG junctions are critical determinants of successful corticogenesis.

This concept is best illustrated in the cortices of mice lacking N-cadherin or other key junctional proteins, including Afadin, αE-catenin, Neogenin, and RhoA (Table 1) (Lien et al., 2006; Kadowaki et al., 2007; Katayama et al., 2011; Cappello et al., 2012; Yamamoto et al., 2013; Gil-Sanz et al., 2014; Schmid et al., 2014; O’Leary et al., 2017; Rakotomamonjy et al., 2017). In these mutants failure of AJ assembly triggers apical RG deadhesion, the collapse of RG morphology and detachment from the ventricular wall. As a consequence, mitotic RGs are no longer restricted to the ventricular surface, but instead redistribute throughout the cortical plate. In addition, retraction of the basal processes prevents radial migration, resulting in neuronal heterotopias.

**TABLE 1:** Apical RG junctional proteins and associated cortical malformations.

**Protein**	**Function at AJs**	**Apical RG phenotype**	**Malformation**
**Junctional proteins**			

N-cadherin	Homophilic cell–cell adhesion, core AJ protein	*Depletion:* loss of AJs and RG morphology, RG detachment from ventricular wall, ectopic RG division, increased RG proliferation, failure of radial migration	SBH^1^ Hydrocephalus
αE-catenin	Tension-sensing molecule, couples N-cadherin/β-catenin to actomyosin		SBH
Afadin	Recruits α-catenin to cadherin at AJs, actin-binding protein		SBH Hydrocephalus

β-Catenin	Required for N-cadherin homophilic adhesion and Wnt signaling, transcriptional activator	*Overexpression*: increased RG proliferation and neuronal production *Depletion:* loss of AJs, premature RG cell cycle exit and neurogenesis	Macrocephaly Heterotopia^2^

**Polarity proteins**			

Par3, aPKC Cdc42	Par3/Par6/aPKC/Cdc42 complex: GTPase, required for AJ formation, establishes RG polarity and identity Par3: stabilizes nascent adhesion sites, promotes symmetric RG division	*Depletion:* loss of AJs and RG morphology, RG detachment from ventricular wall, ectopic RG division, failure of radial migration, premature neurogenesis	aPKC: PVH^3^ Cdc42: heterotopia

Pals	Pals/Crumbs/PatJ complex: maintains RG apicobasal polarity, apical membrane identity, required for AJ formation	*Depletion:* loss of AJs and RG morphology, increased intermediate progenitor production, premature neurogenesis, induction of neuronal cell death	Microcephaly

Lgl, Dlg5	Lgl/Dlg/Scribble complex: maintains RG apicobasal polarity by conferring lateral membrane identity, required for AJ formation, regulates N-cadherin recycling and membrane localization	*Depletion:* loss of AJs and RG morphology, disruption of ventricular wall, ectopic RG division, increased RG proliferation and intermediate progenitor production	Lgl: SBH, PVH Dlg5: hydrocephalus

**Actin regulators**			

RhoA	GTPase, regulator of actomyosin contractility in response to tension, promotes F-actin elongation	*Depletion:* loss of AJs and RG morphology, RG detachment from ventricular wall, ectopic RG division, failure of radial migration	SBH Hydrocephalus
mDia	Promotes F-actin elongation, downstream of RhoA		PVH Hydrocephalus

Cdc42	GTPase, polarity protein, promotes linear actin polymerization, required for AJ formation, establishes RG polarity and identity	*Depletion:* loss of AJs and RG morphology, ectopic RG division, increased RG proliferation and neuronal production	Heterotopia

Myo II	Drives actomyosin contractility and conduction of force across AJs	*Depletion:* loss of AJs and RG morphology, disruption of ventricular wall, failure of radial migration	PVH Hydrocephalus

Arp2/3	Branched actin nucleation at AJs, required for AJ formation and stability	*Arp2, Cyfip1, Abi1 depletion:* depletion of actin cytoskeleton, loss of N-cadherin and other junctional proteins, disassembly of AJs, detachment from ventricular wall, ectopic RG division, premature neurogenesis, attenuation of junctional tension	Heterotopia
WRC	Activates Arp2/3 at AJs, promotes branched actin nucleation		

Neogenin	Recruits WRC and Arp2/3 to AJs, promotes Arp2/3 branched actin nucleation at AJs and actin stability and tension	*Depletion/inhibition of WRC binding:* loss of WRC, Arp2/3 from AJs, depletion of actin and tension at AJs, loss of AJs and RG morphology, disruption of ventricular wall, failure of radial migration, ectopic RG division	SBH, PVH Hydrocephalus

N-WASP	Activates Arp2/3 at AJs, stabilizes F-actin	*Depletion:* inability to form AJs in postnatal ependyma, loss of cilia	Hydrocephalus

**Trafficking proteins**			

FLNA	Regulates N-cadherin trafficking to AJs, actin binding protein	*Depletion/mutations:* loss of AJs and RG morphology, disruption of ventricular wall, failure of radial migration	Human: PVH, hydrocephalus Rodent: PVH

BIG2 *(ARFGEF2)*	Guanine nucleotide exchange factor, regulates N-cadherin trafficking to AJs	*Mutations:* loss of AJs and RG morphology, disruption of ventricular wall, failure of radial migration	Human: PVH, microcephaly Mouse: PVH

αSnap	Regulator of SNARE-mediated vesicular fusion, regulates N-cadherin trafficking to AJs	*Mutations:* failure to deliver N-cadherin to membrane, loss of AJs and RG morphology, disruption of ventricular wall, failure of radial migration	PVH Hydrocephalus

Numb	Localizes N-cadherin-positive recycling endocytic vesicles to AJs, antagonizes Notch to promote neurogenesis	*Depletion:* mislocalization of N-cadherin to apical membrane, loss of AJs and RG morphology, ectopic RG division, failure of radial migration	PVH, Heterotopia Hydrocephalus

*(1) SBH, Subcortical band heterotopia. (2) Heterotopia, abnormal distribution of neurons within cortex. (3) PVH, periventricular heterotopia.*

Cadherin density at the AJ is regulated by vesicular trafficking from the Golgi complex (the biosynthetic pathway) and recycling between internal and plasma membrane pools (Mary et al., 2002; Lock and Stow, 2005; Linford et al., 2012). Disruption of the endosomal system phenocopies loss of N-cadherin. Mutations in the human *FILAMIN-A* (*FLNA*) gene have been identified in inherited and sporadic periventricular heterotopia (Fox et al., 1998; Sheen and Sheen, 2014), whereas periventricular heterotopia can occur with or without microcephaly in patients carrying mutations in the *ARFGEF2* gene which encodes BIG2, a guanine nucleotide exchange factor (Sheen et al., 2003). Both FLNA, an actin binding protein, and BIG2 regulate cadherin trafficking between the Golgi complex and the cell surface. Depletion of FLNA or mutations in *Arfgef2* inhibit AJ assembly and RG polarization in the rodent embryonic cortex resulting in severe disruption of the ventricular surface and the protrusion of nodules into the ventricle (Table 1) (Sheen et al., 2003; Ferland et al., 2009; Carabalona et al., 2012; Sheen and Sheen, 2014). These studies emphasize the importance of maintaining steady-state levels of cadherin at the apical RG junction. In addition, FLNA is known to directly affect the actin cytoskeleton and cytokinesis, and as such, may operate at multiple levels in apical RGs (Sheen and Sheen, 2014).

#### AJ Assembly and Disassembly Govern Corticogenesis

Successful corticogenesis relies on the balance between AJ assembly and disassembly. During symmetric and asymmetric division the acquisition of junctional components allows AJs to reassemble in RG-fated daughters, thereby maintaining polarity and ensuring that they remain integrated into the neuroepithelium. Conversely, commitment to the neural fate is tightly coordinated with the down-regulation of junctional components, allowing detachment from the ventricular surface. For example, at the completion of asymmetric division delamination of the new-born neuron or intermediate progenitor from its RG sister is mandatory for the initiation of radial migration. Upon activation of proneural genes in the neurally-fated daughter, the Scratch transcription factors repress cadherin expression, inducing delamination from the ventricular surface and triggering radial migration (Itoh et al., 2013). When Scratch activity is suppressed cells committed to the neural fate fail to detach and instead accumulate at the ventricular surface. A similar delamination process has evolved in higher order mammals to produce the array of diverse neuronal subtypes underpinning the complexity of the gyrencephalic neocortex. In the ferret the generation of neuronally committed intermediate progenitors, major contributors to cortical expansion, is under precise temporal control (Martínez-Martínez et al., 2016). Within a 2 day embryonic period delamination of the intermediate progenitors is initiated by cadherin down-regulation during asymmetric division. However, increasing cadherin levels prevent intermediate progenitor detachment, a situation which would be predicted to have a profound effect on the development of the gyrencephalic cortex.

Adherens junction assembly is also important for preserving the balance between proliferation and neuronal differentiation. Failure to exit the cell cycle and sustained RG proliferation is a prevalent phenotype observed after genetic deletion of junctional components in apical RGs throughout the embryonic neuroepithelium (Klezovitch et al., 2004; Lien et al., 2006; Katayama et al., 2011; Cappello et al., 2012; Rakotomamonjy et al., 2017; Zhang et al., 2019). The decision to remain an apical RG or exit the cell cycle and adopt a neural fate is under the strict control of environmental proliferative and neurogenic factors that are spatially restricted within the neuroepithelium or present within the CSF (Lehtinen et al., 2011; Vitali et al., 2018; Oberst et al., 2019). In these mutants extensive junctional loss results in the widespread collapse of apical RG morphology. The concomitant withdrawal of the apical end feet and primary cilium from the ventricular surface precludes access to environmental regulatory cues, leading to unrestrained proliferation. This is exemplified by the occurrence of highly proliferative apical RG populations ectopically positioned in the mutant cortical plate which lacks the appropriate regulatory cues.

**FIGURE 2:**
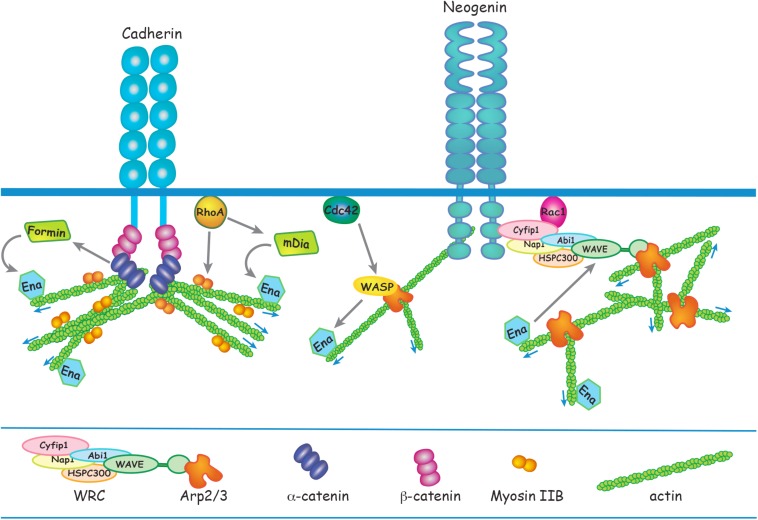
Apical RG AJ assembly is dependent on the reciprocal interactions between cadherins and the actin cytoskeleton. α-catenin links cadherin to F-actin via β-catenin in a tension-dependent manner and also recruits Formin-1 to catalyze linear actin polymerization. RhoA regulates junctional contractility in response to acute tension by promoting phosphorylation of the Myosin regulatory subunit. Ena/VASP adds G-actin to the barbed end of the growing actin filament. N-WASP and mDia act downstream of Cdc42 and RhoA, respectively, to induce Ena/VASP actin polymerization. Branched actin nucleation is initiated at the cadherin adhesion complex by Arp2/3 which is activated by N-WASP or the WRC. Neogenin recruits the WRC and Arp2/3 to cadherin ensuring that branched actin nucleation is confined to the junctional membrane. Ena/VASP binds to WAVE to enhance Arp2/3-mediated branched actin nucleation.

In addition to their non-cell autonomous adhesion function, AJs also influence cell cycle exit in a cell autonomous manner. Stable AJ assembly is dictated by the formation of the core cytoplasmic cadherin-catenin complex comprising β-catenin and α-catenin which link the cadherin to the actin cytoskeleton in a tension-dependent manner (Figure 2) (Drees et al., 2005; Buckley et al., 2014). The interaction between β-catenin and the cadherin intracellular domain is essential for maintaining effective homophilic cadherin binding. β-Catenin is also a key effector of the canonical Wnt pathway that regulates cell cycle exit and cell fate determination throughout the embryo by modulating gene expression. In the absence of Wnt activation, cytoplasmic β-catenin is targeted for degradation by the proteasome. Upon Wnt binding to the Frizzled receptor complex, β-catenin is rescued from degradation and is free to translocate into the nucleus where it activates the transcription of target genes involved in cell fate specification and progenitor proliferation (for detailed reviews see Stocker and Chenn, 2015; Nusse and Clevers, 2017).

During corticogenesis, canonical Wnts are concentrated adjacent to the ventricular surface and thus are perfectly positioned to control the decision to exit the cell cycle. Moreover, β-catenin is expressed highly in apical RGs and is suppressed upon neural commitment (Woodhead et al., 2006; Stocker and Chenn, 2015). Overexpression in RGs of a stabilized form of β-catenin which is able to bind cadherins, but lacks the degradation targeting sites, produces a substantial enlargement of cortical volume due the extensive expansion of the progenitor pool and subsequently excessive neuronal production (Chenn and Walsh, 2002). This dramatic phenotype has been attributed to the inability to exit the cell cycle as a consequence of persistent β-catenin transcriptional activity (Chenn and Walsh, 2002; Stocker and Chenn, 2015). Conversely, depletion of β-catenin triggers early exit from the cell cycle and premature acquisition of neural fate (Machon et al., 2003; Woodhead et al., 2006). In these mutants, AJs fail to form and the proliferative Wnt transcriptional program is silenced. Thus the ability of cadherins to sequester β-catenin at the membrane is likely to be an important modulator of β-catenin transcriptional activity. AJs therefore influence the decision to re-enter the cell cycle by acting as integration sites for cell adhesion and the signaling networks relaying environmental information.

Adherens junctions also influence RG cell fate acquisition through their ability to preserve polarized apical RG morphology. Many studies have demonstrated that multiple interconnected structural factors contribute to apical RG identity, including the retention of the basal process and primary cilium, and the centrosome containing the mother centriole (Konno et al., 2008; Wang et al., 2009; Shitamukai et al., 2011; Wilsch-Brauninger et al., 2012; Paridaen et al., 2013). Establishment of apicobasal polarity, a process dependent on junctional reassembly during cytokinesis, is a prerequisite for retention of these structures. The primary cilium is inherited by the RG-fated daughter and is required for re-entry into the cell cycle, due to its ability to transduce proliferative and morphogenic signals (Eggenschwiler and Anderson, 2007; Oberst et al., 2019). Retention of the primary cilium in the RG-fated cell is dependent on high levels of N-cadherin (Das and Storey, 2014). Conversely, suppression of N-cadherin in the neural daughter by the proneural transcription factor Neurogenin2 prevents the reconstruction of the primary cilium. AJ assembly, therefore, lies upstream of the acquisition of apicobasal polarity and RG cell fate. Induction of RG polarity is governed by multiple polarity complexes whose primary role is to segregate the apical and basolateral membranes. Establishment and maintenance of the polarized state is reliant on the bidirectional interaction between polarity proteins and AJ components.

#### Interplay Between AJs and Polarity Complexes Impose RG Apicobasal Polarity

The defining attribute of all epithelia is their apicobasally polarized morphology which is characterized by compartmentalized apical and basolateral domains comprising distinct repertoires of proteins and organelles. The apicobasally polarized state emerges as a consequence of the synchronized interplay between networks of polarity complexes which act to break symmetry and magnify asymmetry (reviewed in Peglion and Goehring, 2019). A major outcome of polarity network activity is the establishment of AJs which demarcate the border between the apical and lateral membranes. The Par3/Par6/aPKC/Cdc42 polarity complex delineates the apical membrane and opposes Lgl/Dlg/Scribble activity to maintain apical identity (Betschinger et al., 2003; Dollar et al., 2005). Conversely, the Lgl/Dlg/Scribble complex maintains lateral identity by overriding Par complex activity (Yamanaka et al., 2003). Studies in invertebrates and in *in vitro* models (Peglion and Goehring, 2019) have shown that Par3 is localized to nascent sites of cell–cell adhesion and recruits Par6 and aPKC to the adjacent apical membrane. As the epithelial cell matures Par3 dissociates from Par6/aPCK in response to Cdc42-mediated aPKC phosphorylation and relocates to the cadherin adhesion complex, thereby stabilizing the apical domain and emerging AJs (Tanentzapf and Tepass, 2003; Yamanaka et al., 2003). In parallel, exclusion of the Lgl/Dlg/Scribble polarity complex from the apical membrane is achieved through localized deactivation of Lgl by phosphorylated aPKC. Lgl/Dlg/Scribble activity, however, is maintained in the lateral membrane by the pool of non-phosphorylated Lgl which acts to exclude the Par3/Par6 complex from this domain. These spatially restricted interactions reinforce AJ stability and partition the apical and lateral membranes (Betschinger et al., 2003, 2005; Plant et al., 2003; Jossin et al., 2017). During the establishment of polarity the Par3 complex also interacts with Crumbs/Pals/PatJ to reinforce apical identity (Morais-de-Sa et al., 2010; Walther and Pichaud, 2010). Concomitant with the recruitment of Par3 to the AJ, dissociated Par6/aPKC recombine with Pals and Crumbs, a membrane bound receptor confined to the apicolateral membrane. Blocking the Par6-Pals interaction inhibits the redistribution of Par6/aPKC to the apical domain (Hurd et al., 2003).

**FIGURE 3:**
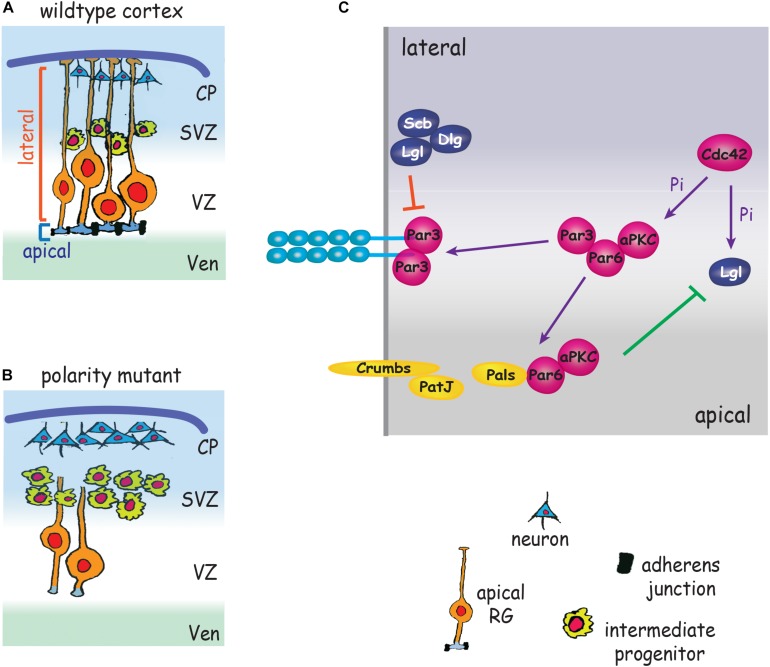
Polarity proteins promote AJ formation and impose RG cell fate. **(A)** Polarity complexes stabilize AJs, promote symmetric RG division and maintain RG polarity by partitioning the apical and lateral membranes. **(B)** Depletion of polarity proteins leads to loss of AJs and RG morphology, RG detachment from the ventricular wall, increased intermediate progenitor production and premature neurogenesis. **(C)** Par3/Par6/aPKC localize adjacent to nascent AJs. Cdc42 induces phosphorylation (Pi) of aPKC. Par3 dissociates from Par6/aPCK and relocates to the cadherin adhesion complex, stabilizing the apical domain and emerging AJs. Par6/aPKC recombine with Pals/Crumbs/PatJ to define the apical membrane. aPKC also phosphorylates Lgl, leading to exclusion of Lgl/Dlg/Scribble from the apical membrane (green line). Non-phosphorylated Lgl excludes Par3/Par6/aPKC from the lateral membrane (red line). CP, cortical plate; SVZ, subventricular zone; Ven, ventricle; VZ, ventricular zone.

As in all epithelia, the relationship between RG polarity proteins and AJs is bidirectional. Initiation of junction assembly is dependent on polarity protein signaling, and consolidation of the polarized state requires that AJ components confine polarity proteins to the appropriate membrane compartment. The Par3/Par6/aPKC/Cdc42, Lgl/Dlg/Scribble, and Crumbs/Pals/PatJ polarity complexes play pivotal roles in promoting RG apicobasal polarity and AJ formation (Figure 3 and Table 1), and as a consequence, are key factors in determining progenitor fate. However, whether AJs play an instructive role in regulating cell fate commitment and cell cycle exit independently of their role in adhesion remains an open question.

Direct evidence that the Par complex and AJs collaborate in imposing the apical RG phenotype is patently demonstrated by deletion of the polarity determinants aPKC, Par3 or the small GTPase Cdc42, a Par complex activator (Cappello et al., 2006; Imai et al., 2006; Bultje et al., 2009). Loss of these polarity proteins promotes AJ failure, the retraction of the RG basal process and the withdrawal of the apical end feet from the ventricular surface (Figure 3). As a result RGs undergo precocious differentiation into intermediate progenitors and neurons (Cappello et al., 2006; Bultje et al., 2009). The inhibition of AJ-Par complex interactions therefore promotes the adoption of the neurogenic fate at the expense of the proliferative RG fate, leading to premature neurogenesis. As a consequence of the imbalance in cell fate specification, inadequate numbers of progenitors are generated early in corticogenesis, resulting in long-term depletion of neuronal populations, a phenotype underpinning the etiology of microcephaly (Pinson et al., 2019). This also appears to be the *modus operandi* of Zika virus which causes microcephaly by preferentially infecting apical RGs (Yoon et al., 2017). Zika proteins interact with multiple AJ components resulting in impaired AJ assembly, suppression of RG proliferation and premature neurogenesis in the mouse cortex and in human iPSC-derived organoids.

The Pals polarity protein links Crumbs/PatJ to the Par3/Par6/aPKC complex which act together to define the apical membrane domain. Therefore, it is not surprising that genetic deletion of *Pals* in apical RGs also has devastating consequences for cortical development (Kim et al., 2010). As seen in the *Par3* mutant, apical RGs lacking Pals undergo premature cell cycle exit and extensive neuronal differentiation early in corticogenesis. In the absence of Pals, Crumbs and aPKC no longer associate with the junctional complex, leading to AJ disassembly or a basal shift in AJ location, and the abrogation of RG apicobasal polarity. However, in contrast, to the *Par3* mutant, a rapid induction of neuronal cell death was observed in late cortical development due to the inhibition of the mTOR cell survival pathway.

Inhibition of the Lgl/Dlg/Scribble polarity complex also has profound effects on cortical development. Genetic depletion of Lgl prevents AJ formation and RG polarization and disrupts the ventricular wall (Klezovitch et al., 2004; Beattie et al., 2017; Jossin et al., 2017; Zhang et al., 2019). Both periventricular and subcortical heterotopias are observed in these mutants. Failure to exit the cell cycle translated into excessive RG proliferation and increased neurogenesis, further exacerbating the subcortical heterotopia phenotype. In the absence of Lgl a substantial decrease in N-cadherin endocytosis was observed (Jossin et al., 2017). *In vitro* and *in vivo* experiments revealed that N-cadherin internalization and AJ stabilization require a direct interaction between Lgl and N-cadherin, and that this interaction is inhibited by aPKC-mediated phosphorylation of Lgl. Together, these findings suggest a model whereby Lgl triggers N-cadherin endocytosis at the lateral membrane, but is prevented from doing so at the AJ due to inhibition by junctionally localized aPKC. As a result, high levels of N-cadherin are confined to the junction. This hypothesis is bolstered by a previous study demonstrating that the deletion of aPKC in the embryonic cortex also prevents AJ formation and the establishment of polarized RG morphology (Imai et al., 2006). The central role of the Lgl/Dlg/Scribble complex in the regulation of cadherin recycling at RG junctions is further supported by *Dlg5* loss-of-function embryos which exhibit reduced membrane N-cadherin and phenocopy the *Lgl* mutants (Nechiporuk et al., 2007). *In vitro* studies show that the efficient delivery of cadherin containing vesicles to the membrane relies on the interaction between Dlg5 and Syntaxin4, a member of the q-SNARE membrane fusion complex as well as between Dlg5 and the junctional component, β-catenin (Nechiporuk et al., 2007). These data lead to the hypothesis that Dlg5 anchors the vesicular fusion machinery to AJs to reinforce apical RG junctional integrity.

Adherens junctions also influence the decision to re-enter the cell cycle by spatially coordinating extrinsic signals with polarity complex activity. The stem cell determinant Notch is localized to the apical RG junctional membrane where it associates with the cadherin adhesion complex. AJ assembly is required for Notch activation and retention of the RG fate by sequestering Par3 during asymmetric division (Del Bene et al., 2008; Bultje et al., 2009; Ohata et al., 2011; Hatakeyama et al., 2014). Notch functions downstream of Par3 to specify progenitor identity, whereby the daughter cell inheriting the greatest proportion of Par3 exhibits high Notch activity. Overexpression of Par3 forces cells to adopt the apical RG fate due to the enrichment of Par3 and Notch activity in both daughter cells, whereas Par3 suppression deactivates Notch and confers neuronal identity to both cells. Intriguingly, several studies have revealed an explicit link between Notch signaling and cadherin-mediated adhesion. Numb, a known regulator of Notch clathrin-mediated endocytosis, promotes the acquisition of neuronal cell fate during RG asymmetric division by antagonizing Notch signaling (Rasin et al., 2007; Bultje et al., 2009). Moreover, Numb is found in the apical end feet at interphase where it specifically localizes to cadherin-positive recycling endocytic vesicles and directly interacts with cadherins (Rasin et al., 2007). Silencing Numb activity results in mislocalization of cadherin to the RG apical membrane and prevents junctional assembly, leading to loss of RG morphology, ectopic positioning of neurally fated cells and aberrant cortical lamination. Conversely, overexpression of Numb provokes persistent apical RG anchoring to the ventricular surface, sustained apicobasal polarity and retention of progenitor identity in both siblings. These observations provide persuasive evidence that junction assembly and cell fate determination are tightly coordinated.

#### Do AJs Influence the Mode of Cell Division?

During symmetrical proliferative divisions retention of junctions and the associated polarity proteins promotes re-establishment of apicobasal polarity and confers RG progenitor identity, whereas depletion of these factors after asymmetric division bestows neural fate. Pioneering studies have revealed that the inheritance of the Par complex and junctional components is dependent on the position of the cleavage plane during cytokinesis (Huttner and Kosodo, 2005; Konno et al., 2008; Marthiens and Ffrench-Constant, 2009; Paridaen et al., 2013). In the early phase of corticogenesis when progenitors are undergoing symmetric self-renewing divisions, mitotic spindles are orientated in the planar orientation (vertical cleavage plane). Cleavage along the vertical plane bisects the apical membrane resulting in the equal distribution of junctional and polarity proteins between the daughters, thereby promoting the proliferative RG fate. As apical RGs switch to asymmetric neurogenic divisions, the mitotic spindle re-orientates such that the cleavage plane becomes horizontal or oblique, favoring the production of neurons or intermediate progenitors (Huttner and Kosodo, 2005; Konno et al., 2008; Postiglione et al., 2011; Paridaen and Huttner, 2014). In this situation retention of the RG fate is associated with the acquisition of the greater fraction of AJ components and polarity proteins. Conversely, the exclusion of these factors promotes commitment to the neurogenic fate. Whether or not AJs directly influence spindle orientation remains controversial. However, increased oblique divisions and a subsequent increase in intermediate progenitors and their neuronal offspring have been reported after deletion of the junctional component Afadin (Rakotomamonjy et al., 2017), suggesting that AJ component inheritance favors the apical RG fate and inhibits asymmetric division. This premise is supported by studies in other epithelia where Afadin binding to F-actin and LGN, a microtubule motor regulator of spindle orientation, promotes symmetric proliferative division (Carminati et al., 2016). Thus, junctional stability, apicobasal polarity and the direction of cytokinesis are intimately linked.

### The Actin Cytoskeleton Fortifies Apical RG Adherens Junctions

In the embryonic brain AJs must resist the mechanical stresses exerted by dynamic cellular behaviors and increased CSF pressure. As in other epithelial tissues the maintenance of apical RG morphology and neuroepithelial integrity relies on the adhesive strength dictated by reciprocal interactions between the cadherins and the circumferential actin ring which runs parallel to the AJ within the RG apical end feet (Figure 1). Adhesive strength is dependent on the continual turnover of the actin cytoskeleton, and actomyosin contractility regulates junctional tension and thus resilience to stress (Maître et al., 2012; Priya and Yap, 2015; Yap et al., 2017). Failure to remodel the actin cytoskeleton in apical RGs leads to AJ disassembly, loss of polarized morphology and perturbation of the neuroepithelial cytoarchitecture. The fundamental role of actin remodeling in maintaining polarized RG morphology and neuroepithelial integrity is reinforced by the emergence of cortical malformations in mice lacking actin regulators (Table 1).

Spatiotemporal regulation of actin polymerization is coordinated through the Rho GTPases RhoA, Rac1, and Cdc42 which govern actin remodeling by directly linking a diverse array of actin modulators to cell surface receptors and downstream effectors (Figure 2) (Priya and Yap, 2015; Lee et al., 2016; Braga, 2017; Acharya et al., 2018; Schaks et al., 2018). RhoA is a major regulator of junctional actomyosin contractility and is also recruited to AJs in response to acute tension, thereby reinforcing the actin cytoskeleton (Priya and Yap, 2015; Acharya et al., 2018). Conditional deletion of RhoA in apical RGs inhibits incorporation of G-actin into the F-actin filament, leading to the destabilization of the actin cytoskeleton and AJ disassembly (Cappello et al., 2006; Katayama et al., 2011). Subsequent RG detachment from the ventricular surface leads to ectopic positioning above the ventricular zone. In addition, although loss of RhoA was found not to affect neuronal motility *per se*, neurons were unable to undergo radial migration due to the retraction of the RG basal processes. Instead, neurons accumulated under the cortical plate forming subcortical band heterotopias. RhoA activation of formins such as mDia promotes F-actin elongation (Figure 2). Therefore, it is not surprising that the ablation of the RhoA effector mDia in RGs phenocopies the RhoA mutants, whereby junctional disruption provokes neuronal heterotopias (Thumkeo et al., 2011).

The importance of a direct link between cadherin and the RG actin cytoskeleton is further demonstrated by the conditional deletion of αE-catenin (Lien et al., 2006; Schmid et al., 2014). In epithelial tissues the assembly of functional AJs is dependent on the interaction between cadherin and the cytoplasmic effector, α-catenin which couples the cadherin cytoplasmic domain (via β-catenin) to the actin cytoskeleton (Figure 2) (Drees et al., 2005; Buckley et al., 2014). Loss of αE-catenin in apical RGs induces AJ disassembly due to the inability to generate a stable actin cytoskeleton, leading to the retraction of the basal process which in turn severely affects radial migration. Here clusters of neurons accumulate below the cortical plate forming a second neuronal layer equivalent to that seen in subcortical band heterotopia. Insight into the mechanisms underpinning αE-catenin function at RG junctions comes from *in vitro* experiments using optical trap-based assays to quantify α-catenin-actin binding under applied force (Buckley et al., 2014). These experiments revealed that α-catenin is a tension-sensing molecule as it is only able to couple F-actin to cadherins when force is applied (Buckley et al., 2014). It is likely, therefore, that αE-catenin acts in a similar manner at RG junctions, thereby providing a sensitive mechanism with which to detect and modulate junctional tension at the ventricular surface.

Actomyosin activity is dynamic and responds to forces experienced at the junction by modulating cadherin adhesion and promoting actin remodeling, thereby ensuring AJ stability (Smutny et al., 2011; Borghi et al., 2012; Priya and Yap, 2015; Yap et al., 2017). Myosin II drives actomyosin contractility at AJs and therefore plays a central role in the regulation of junctional tension. Upon myosin binding to the actin filament, RhoA-dependent phosphorylation of the myosin regulatory subunit triggers contractility (Figure 2). Coupling of myosin to the cadherin cytoplasmic domain enables conduction of force across the junction and facilitates the transmission of actomyosin-induced tension across cell ensembles (Smutny et al., 2011; Borghi et al., 2012; Priya and Yap, 2015). Early pioneering studies using genetic deletion of Myosin IIB provide compelling evidence that actomyosin activity plays an essential role in maintaining apical RG junctional integrity and neuroepithelial cohesion (Tullio et al., 2001; Ma et al., 2007). As seen in N-cadherin loss-of-function mutants, deletion of Myosin IIB leads to AJ failure, collapse of apical RG morphology and disruption of the ventricular surface throughout the neuraxis. Within the forebrain, aggregations of neurons and progenitors protrude into the ventricular space, a phenotype reminiscent of the periventricular heterotopias seen in humans. Furthermore, actomyosin contractility has been shown to play a central role in promoting the abscission of new-born neurons from the ventricular surface during asymmetric RG division, a process required for the initiation of radial migration (Das and Storey, 2014). This activity lies downstream of N-cadherin as increased N-cadherin or inhibition of myosin activity prevents abscission of the prospective neuron.

A common feature of mutants lacking key actin regulators (αE-catenin, RhoA, Myosin IIB, Arp2/3), is the occurrence of apical RG rosettes within the cortical plate where clusters of polarized progenitors assemble around a central lumen (Tullio et al., 2001; Lien et al., 2006; Kadowaki et al., 2007; Katayama et al., 2011; Cappello et al., 2012; Schmid et al., 2014; Wang et al., 2016). The subapical membrane localization of cadherin, catenin, and Par3 preserves cohesion despite the depletion of junctional actin. This is in stark contrast to the ventricular wall where AJ stability in the RG apical end feet is clearly diminished, indicating that in the absence of the actin regulators junctional adhesive strength is manifestly attenuated and unable to buffer the forces exerted at the ventricular surface. The mechanical stresses experienced within the cortical plate, however, would be substantially lower, allowing the compromised junctions to support adhesion. These observations highlight the critical role played by the actin cytoskeleton and its network of regulatory proteins in reinforcing tissue cohesion while preserving junctional flexibility in the embryonic neuroepithelium.

### Branched Actin – The Epicenter of the Junctional Cytoskeleton

Investigations using *in vitro* epithelial cell models have revealed that the junctional actin cytoskeleton consists of a dense network of branched actin which seeds the growth of the linear F-actin filaments comprising the actin ring (Verma et al., 2012; Brieher and Yap, 2013; Han et al., 2014; Lee et al., 2016). Branched actin nucleation is initiated at the cadherin adhesion complex and is mediated by the Arp2/3 enzymatic complex, an inefficient actin nucleator (Pizarro-Cerda et al., 2017). Arp2/3 is activated by the pentameric WAVE regulatory complex (WRC), a pivotal branched actin regulator composed of five subunits organized into the Cyfip/Nap and WAVE/Abi/HSPC300 subcomplexes (Figure 2) (Chen et al., 2010, 2014). Arp2/3 activity is triggered upon binding to the WAVE subunit, and this interaction is dependent on activated Rac1 binding to Cyfip1 within the holocomplex (Chen et al., 2014; Schaks et al., 2018). Both Arp2/3 and the WRC are tightly associated with AJs and play an important role in the epithelial response to mechanical stress as their dissociation from the cadherin adhesion complex induces junctional disassembly and the attenuation of junctional tension (Verma et al., 2012; Han et al., 2014; Lee et al., 2016).

In RG junctions branched actin nucleation is also dependent on Arp2/3 and the WRC, both of which are concentrated within the RG apical end feet (Yoon et al., 2014; Wang et al., 2016; O’Leary et al., 2017). Conditional deletion of the obligatory Arp2 subunit in the mouse embryonic cortex results in depletion of the actin cytoskeleton from the subapical membrane, loss of N-cadherin and other key junctional proteins and disassembly of AJs (Yoon et al., 2014; Wang et al., 2016). As a consequence of junctional failure, RGs detach from the ventricular surface and form ectopic rosettes in the cortical plate. Moreover, those RGs remaining at the ventricular surface undergo premature commitment to the neuronal fate, which ultimately leads to a decrease in mature pyramidal populations. Similarly, depletion of the WRC subunits Abi1 or Cyfip1 results in the loss of AJs and polarized RG morphology with concomitant ectopic RG redistribution to the upper cortical layers and marked perturbation of the neuroepithelium (Yoon et al., 2014).

Branched actin nucleation precisely at the AJ requires that the WRC and Arp2/3 is spatially restricted to the cadherin complex. A critical molecular mechanism responsible for the recruitment of Arp2/3 and the WRC to cadherins has recently been discovered. Neogenin, a multi-functional receptor for Netrin and the Repulsive Guidance Molecules (Rajagopalan et al., 2004; De Vries and Cooper, 2008; Kee et al., 2008; O’Leary et al., 2015; Siebold et al., 2017), has been identified as a pivotal component of the branched actin nucleation machinery governing junctional stability and tension (Lee et al., 2016; O’Leary et al., 2017). The WRC interacting receptor sequence (WIRS), found in the intracellular domain of Neogenin, binds to a highly conserved binding pocket within the WRC composed of the Cyfip1 and Abi subunits (Figure 2) (Chen et al., 2014; Lee et al., 2016). Using an *in vitro* epithelial model it was demonstrated that Neogenin recruitment of the WRC to the cadherin adhesion complex ensures that Arp2/3 actin nucleation is confined precisely to the junctional membrane, thereby promoting actin ring stability and junctional integrity. Loss of Neogenin severely perturbs AJ integrity due to the attenuation of junctional tension resulting from fragmentation of the actin ring (Lee et al., 2016). As would be predicted, blocking the interaction between Neogenin and the WRC subunits Cyfip1 and Abi in RGs prevents Arp2/3-mediated actin nucleation, leading to the loss of F-actin, disintegration of the actin ring and AJ destabilization (O’Leary et al., 2017). The ensuing retraction of RG basal processes and disruption of the ventricular wall prevents radial migration into the cortical plate, resulting in the emergence of periventricular and subcortical band heterotopias.

The activity of the branched actin nucleation machinery and tension-sensing factors are coordinated to ensure that actin remodeling is responsive to local forces generated within the epithelium. Biallelic mutations in the human αN-catenin gene *CTNNA2* is causative for a recessive form of pachygyria (reduction in gyrus formation) (Schaffer et al., 2018). This study and an earlier biochemical study (Drees et al., 2005) revealed that in addition to its role in coupling linear actin to the cadherin complex, α-catenin is able to regulate branched actin polymerization by competing with Arp2/3 for G-actin binding in migrating pyramidal neurons. This competitive behavior is dependent on high concentrations of free cytoplasmic α-catenin, a situation that is likely to arise as AJs mature and cadherin-α-catenin interactions become saturated. As α-catenin also recruits formins to catalyze the polymerization of linear actin filaments at AJs (Kobielak et al., 2004), this suggests that α-catenin may modulate junctional tension by balancing the synthesis of branched and linear actin and that perturbation of this regulatory mechanism is likely to impact junctional stability. We would therefore speculate that αE-catenin functions in a similar manner to stabilize RG junctions.

Branched actin nucleation and linear elongation factors work in unison to reinforce the bidirectional interplay between the cadherin adhesion complex and the actin cytoskeleton. Actin nucleation is the rate-limiting step for polymerization of both branched and linear actin. N-WASP, like its close relative WAVE, is an Arp2/3 activator (Figure 2) (Rohatgi et al., 1999). It also functions to stabilize linear actin filaments at AJs and in doing so sustains contractile tension (Kovacs et al., 2011; Wu et al., 2014). The elongation factor Ena/VASP promotes the addition of G-actin to the barbed end of the growing actin filament (Siton-Mendelson and Bernheim-Groswasser, 2017). N-WASP and the formin mDia act downstream of Cdc42 and RhoA, respectively, to induce Ena/VASP-mediated growth of linear actin from branched nodes (Figure 2). Silencing this signaling pathway in the neuroepithelium by deleting N-WASP, mDia, RhoA or Cdc42 interferes with junctional integrity, leading to cortical malformations (Symons et al., 1996; Cappello et al., 2006; Katayama et al., 2011; Thumkeo et al., 2011; Cappello et al., 2012; Jain et al., 2014). In addition, Ena/VASP has been shown *in vitro* to bind to WAVE and potentiate Arp2/3-mediated branched actin nucleation (Havrylenko et al., 2015). Together, these observations implicate the WAVE/WASP proteins as a central signaling hub at RG junctions that acts to ensure synergistic linear and branched actin polymerization in order to modulate junctional tension and maintain tissue cohesion.

### Adherens Junctions, the Ependymal Epithelium and Hydrocephalus

Ependymal cells form a multi-ciliated cuboidal epithelial monolayer that acts as a bidirectional barrier between the ventricle and the brain parenchyma, and the establishment of an apicobasally polarized ependyma is essential for the maintenance of brain homeostasis (Figure 1D) (Del Bigio, 2010; Jimenez et al., 2014). The vectoral transport of proteins, nutrients and waste products across the ependymal epithelium is driven by transporters and channel proteins which are asymmetrically distributed at the apical membrane facing the CSF or the basal membrane adjacent to the parenchyma. In addition, a polarized epithelium is necessary for the elaboration of the motile cilia on the apical surface which propel CSF through the ventricular system. Loss of ependymal integrity resulting from progressive ventricular denudation beginning early in fetal life is the underlying pathology in some forms of perinatal hydrocephalus (Dominguez-Pinos et al., 2005; Rodriguez et al., 2012; Guerra et al., 2015). Denudation of the ependymal epithelium severely hampers nutrient and toxin exchange, and the absence of a multi-ciliated epithelium directly impacts CSF flow further exacerbating ventricle dilation.

Ependymal cells are derived from the apical RGs of the embryonic neuroepithelium (Ortiz-Álvarez et al., 2019; Redmond et al., 2019). In the human embryo, RG expansion and neurogenesis occur between gestational weeks 12 and 22 (Jimenez et al., 2014; Guerra et al., 2015). In parallel, a subpopulation of RGs differentiate into ependymal cells with the mature ependyma being established by gestational week 28. The assembly of resilient AJs is critical to the integrity of the ependymal epithelium which must resist the tensile forces arising from increasing CSF volume and other mechanical stresses associated with the expansion of the ventricles in the embryo and neonate. Evidence is now accumulating that denudation and thus hydrocephalus originate in the embryonic neuroepithelium prior to the onset of ependymal maturation due to the failure of junctional assembly in apical RGs and subsequently ependymal cells (Figure 1E) (Jiménez et al., 2009; Rodriguez et al., 2012; Guerra et al., 2015). Moreover, the occurrence of hydrocephalus is often coincident with neuronal heterotopias. For example, periventricular heterotopias are accompanied by the disruption of the ependymal epithelium in postmortem brains of patients carrying *FLNA* mutations (Sheen et al., 2004; Ferland et al., 2009; Jimenez et al., 2014; Lian and Sheen, 2015). Thus hydrocephalus and some forms of cortical malformations share a common etiology – the loss of AJs.

This hypothesis is unequivocally supported in mouse models in which the molecular pathways governing AJ assembly have been disrupted. In the embryonic mouse cortex apical RGs are specified as ependymal cells between embryonic days 14 and 16 (Ortiz-Álvarez et al., 2019; Redmond et al., 2019). However, in contrast to the human, committed apical RGs do not transform into a cuboidal ependymal epithelium until the first week of postnatal life (Tramontin et al., 2003; Spassky et al., 2005). By the end of the second postnatal week all RGs have terminally differentiated into ependymal cells. Hydrocephalus occurs in mice lacking AJ-associated proteins, such as N-cadherin, Yap, Afadin, mDia, Dlg5, Numb, or Plekha7 (Nechiporuk et al., 2007; Rasin et al., 2007; Thumkeo et al., 2011; Oliver et al., 2013; Yamamoto et al., 2013; Park et al., 2016; Tavano et al., 2018), all of which control cadherin adhesion in the apical RG end feet. In addition, disruption of cadherin trafficking has profound effects on both neuroepithelial and ependymal integrity. A mutation in *αSNAP*, a regulator of SNARE-mediated vesicular fusion, has been identified in the hydrocephalus mouse model, *hyh* which also exhibits periventricular heterotopia (Chae et al., 2004; Paez et al., 2007; Ferland et al., 2009; Jiménez et al., 2009). In these mice the failure to deliver cadherin to the plasma membrane prevents AJ formation, leading to severe ventricular disruption from early corticogenesis onwards.

As described above, depletion of the branched actin regulators Neogenin, the WRC and Arp2/3 induces cortical heterotopias due to the inability to establish stable RG junctions. In addition, genetic depletion of Neogenin results in extensive denudation of the ventricular wall by postnatal day 3 and ongoing loss of mature ependymal cells throughout the adult ventricular system, resulting in severe hydrocephalus (O’Leary et al., 2017). *In vitro* and *in vivo* studies confirmed that blocking Neogenin-WRC interactions prohibits the formation of both RG and ependymal junctions. These studies highlight the pivotal role played by the Neogenin-WRC-Arp2/3 actin regulatory network in promoting actin remodeling, AJ stability and the integrity of the embryonic neuroepithelium and the postnatal ependymal epithelium. The fundamental role of actin in maintaining the fidelity of both these epithelia is further reinforced by the emergence of neuronal heterotopias and hydrocephalus in mice lacking actin modulators, including RhoA, Myosin IIB, mDia, and N-WASP (Tullio et al., 2001; Ma et al., 2007; Thumkeo et al., 2011; Jain et al., 2014).

## Conclusion

We now have a robust understanding of the key interactions between junctional components and the epithelial polarity complexes which govern RG apicobasal polarity as well as the essential molecular networks promoting AJ assembly and stability (e.g., actin regulators). Nonetheless, we have a limited understanding of how the activity of these many AJ interactors are spatiotemporally coordinated at the junctional interface in the steady state and in response to environmental stresses. Clearly there is a large number of proteins competing for binding sites at the RG junction. However, we currently have little insight into the nanodomain organization of these protein complexes. Do the cadherins form large multi-protein complexes or do they form discrete nanodomains comprising one or a few proteins? Is the mobility of these nanodomains coordinated in time and space? How are these protein–protein interactions synchronized at the nanoscale level in response to regulatory signals and what are the kinetic principles driving these interactions? With the advent of powerful super-resolution microscopic technologies such as dSTORM (stochastic optical reconstruction microscopy) and sptPALM (single-particle tracking photoactivated localization super-resolution microscopy) we are now in a position to address these intriguing and important questions.

It was not until the late 1980s and early 1990s that apical RGs were recognized as the neural stem cells responsible for the generation of all pyramidal neurons of the mammalian cortex (Misson et al., 1988; Malatesta et al., 2000; Hartfuss et al., 2001). Prior to this, RGs were thought to be a fully committed, non-dividing astroglial cell type derived from a lineage distinct from that giving rise to neurons. As such, apical RGs were believed to play only a supportive role in the developing cortex. This review provides a glimpse into the tremendous progress that has been made over the past 30 years in unraveling the fundamental, multifaceted role of apical RGs in the development of the neocortex. As underscored here, AJs play an obligate role in the establishment of RG apicobasal polarity and hence function. The fidelity of cortical development is therefore critically dependent on AJ assembly and stability. This is overwhelmingly illustrated by the occurrence of debilitating cortical malformations, including perinatal hydrocephalus, arising as a consequence of junctional failure.

## Author Contributions

LV: this review is part of LV’s Ph.D. thesis Introduction. He contributed to the intellectual content, selection of review content, interpretation of the literature, and manuscript preparation. CO: contributor to the intellectual content, interpretation of the literature, and manuscript preparation. He also provided content for the figures. HC: senior author and major contributor to the intellectual content, including selection of review content and interpretation of the literature. She also contributed to manuscript preparation.

## Conflict of Interest

The authors declare that the research was conducted in the absence of any commercial or financial relationships that could be construed as a potential conflict of interest.
